# Antibiotic use in urological surgeries: a six years review at Muhimbili National Hospital, Dar es salaam-Tanzania

**DOI:** 10.11604/pamj.2015.22.226.6253

**Published:** 2015-11-11

**Authors:** Obadia Nyongole, Larry Akoko, Ally Mwanga, Mabula Mchembe, Benjamin Kamala, Naboth Mbembati

**Affiliations:** 1 Department of Surgery, School of Medicine, Muhimbili University of Health and Allied Sciences, Tanzania; 2 Department of community Medicine, Hubert Kairuki Memorial University, Dar es Salaam, Tanzania

**Keywords:** Antibiotics, urological surgeries, Tanzania

## Abstract

**Introduction:**

Antimicrobial prophylaxis for urologic procedures is a major issue, as potential advantages of antibiotic administration should be carefully weighed against potential side effects, microbial resistance, and health care costs. This study aimed to review a six years trend of antibiotic use in urological surgeries at Muhimbili National Hospital (MNH) being an experience in a typical third world environment.

**Methods:**

This was a six years hospital based descriptive, retrospective study conducted of which all case notes of urological patients operated on in between January 2007 to December, 2012 were reviewed by using a structured data collecting tool. The data were analyzed using SPSS software.

**Results:**

Male patients were the majority at 62% (450). The age range was 0 - 90 years, with a mean of 30 ± 22.09. Among the urological surgeries done at MNH 86.5% (628) received prophylactic antibiotics regardless of the type surgery done. Majority 63.7% (463) received antibiotics during induction. Ceftriaxone was the commonly given antibiotic regardless of the type of urological surgery done. Most of patients (86.4%) were given antibiotics for five days regardless whether it was for prophylactic or treatment intention.

**Conclusion:**

Antibiotic use is still a challenge at our hospital with over use of prophylactic antibiotics without obvious indications. Prolonged use of prophylactic antibiotics beyond five days was the main finding. Ceftriaxone was the most given antibiotic regardless of the urological surgery done and its level of contamination. Antibiotic stewardship needs to be addressed urgently to avoid serious drug resistances leaving alone the cost implication.

## Introduction

Antibiotics are synthetic molecules that can destroy or inhibit the growth of microorganisms without harming the host. They can be used for prophylactic purposes to reduce the incidence of postoperative infection of which duration should not exceed 24hrs in many procedures given one hour prior to incision. Also antibiotics for treatment purposes are given when an established infection has been identified.Antibiotic prophylaxis is a brief course of antibiotics administered before or at the start of an intervention and used to minimize the infectious complications resulting from diagnostic and therapeutic interventions. While the rationale for the use of antibiotics is well accepted, possible side-effects and development of microbial resistance patterns are potential risks. Therefore, an antibiotic prophylaxis policy should be well considered and, ideally, based on high levels of evidence. Urology is a surgical speciality which has under-gone many changes in the last decade. Surgical procedures have mainly shifted from open to endoscopic and laparoscopic procedures, and nowadays, a greater number of elderly patients or carriers of temporary urinary derivations are being operated on. These developments can influence the choice of antibiotic prophylaxis policy. Although it is common practice to administer antibiotic prophylaxis in many urologic procedures, there is still little evidence for the use of antibiotic prophylaxis in most of these procedures. This is mainly due to the lack of well-designed studies as well as the lack of clear definitions of favourable outcome parameters. The question remains to what extent antibiotic prophylaxis is beneficial in the different urologic procedures. Various authors have addressed this issue in reviews in recent years [1–5]. Also, the European Association of Urology (EAU) has recently updated the guideline “Management of urinary and male genital tract infections”, including a chapter on perioperative antibacterial prophylaxis in urology. However with the exception of the transurethral resection of the prostate (TURP) [6–14], some of the recommendations in these reviews and guidelines are supported by evidence gathered in a structured systematic review. Surgical wound classification in the categories clean, clean-contaminated, contaminated and dirty seems just as relevant for urologic surgery as for general surgery [15, 16]. In this way assessing the pre-intervention surgical wound class, an estimate can be made of the need for antibiotic prophylaxis during surgery. Clean surgery involves uninfected tissues without opening of the urinary tract and with primary closure of the wound. In clean contaminated surgery, the urinary tract is entered under controlled conditions, without the presence of infected tissues or bacteriuria. Surgery with use of bowel tissue is also classified as clean-contaminated. The presence of a non treated infection, including UTI, should be considered as contaminated urologic surgery. When pus is present, the surgery is labelled dirty. Implantation of prosthesis material is not classified as above. Since infectious complications are potentially serious when involving prosthesis material, antibiotic coverage is advocated irrespective of surgical class [2, 12, 16]. Derived from the surgical literature and not supported by urologic evidence, there is no indication for antibiotic prophylaxis in clean surgery, whereas there is an indication in clean-contaminated and prosthetic surgery. Contaminated and dirty surgery should be covered by therapeutic antibiotics instead of prophylactic dosages. Our study aimed to review a six years trend of antibiotic use in urological surgeries at Muhimbili National Hospital (MNH) being an experience in a typical third world environment.

## Methods

This was a retrospective study at Muhimbili National Hospital (MNH)- medical record department from November 2013 to April 2014. All case notes of urological patients operated on in between January 2007 to December, 2012 was included in the study.

### Inclusion criteria

All available case notes of operated patients in urology unit during the period of 2007 to 2012 were eligible for use in this study.

### Exclusion criteria

Case notes with incomplete information were not considered in some of the analysis where such missing variable are imperative.

### Data processing and analysis

A structured questionnaire was used to collect data from patient's case notes. An electronically generated medical detail of all operated patients was obtained per year. The details that were possible to obtain included the age, sex, file number, date of operation and type of operation. From this, patients who were operated under urology unit were identified and their case notes retrieved. Data coding was done, cleansed and entered into computer software for storage and analysis using SPSS version 18 statistical program. Frequencies were run for all categorical variables and patterns of surgery and their outcome described. Mean and standard deviation were used to summarize continuous variables.

### Ethical issues

Ethical clearance to conduct the study was obtained from the Institutional Review Board (IRB) at Muhimbili University of Health and Allied Sciences (MUHAS) **REF.No.MU/DRP/AEC/Vol.XVIII/22 of 30th October 2013** and a separate permission to conduct the study was also obtained from the Executive Director of Muhimbili National Hospital (MNH). Direct patient identifiers were not used except for file numbers during initial data collection; thereafter the questionnaires were coded for further analysis.

### Study limitations

This study was done at Muhimbili National Hospital which is the only national referral hospital located in the Centre of Dar es Salaam city, thus the findings may reflect a true image of the trend of antibiotic use in urological surgeries in Dar es Salaam and country. At large but incomplete documentation might impair the image.

## Results

A total 726 case notes of patients who underwent urological surgeries during the study period of six years were reviewed. The age range was 0 - 90 years, with a mean of 30 ± 22.09 with male predominance giving a ratio of 6.4: 1. Cystoscopy 260 (36%) was the most commonly done urological surgery followed by prostatectomy 154 (21%) (Table 1). Most of our patients 589 (81.1%) were given antibiotics regardless of the type urological surgeries. Eighty seven point four percent of clean contaminated urological surgeries were given prophylactic antibiotics also those who had clean urological surgeries 265 (74.6%) were given antibiotics. There were neither contaminated nor dirty urological surgeries done (Table 2). Majority of our patients 63.7% (463) received antibiotics during induction. The commonly given antibiotics were Ceftriaxone combined with metronidazole 37.5% and ceftriaxone alone was given in 46.1% with Gentamycin being given alone in 6% only. Ceftriaxone was the commonly given antibiotic regardless of the type of urological surgery done (Table 3).

**Table 1:** The distribution of urological surgeries

Procedure	Number	Percentage
Cystoscopy	260	36%
Prostatectomy	154	21%
Urethroplasty	113	16%
Nephrectomy	61	8%
Hyospadias repair	43	5.9%
Others(DVU, TCB, BSO, SPC, Cystectomy)	95	13.1%
Total	726	100

DVU (Direct Vision Urethrotomy), TCB (Tru-Cut Biopsy of prostate), BSO(Bilateral Sub capsular Orchiectomy), SPC(Suprapubic Cystostomy)

**Table 2:** Levels of contamination in urological surgeries and antibiotic use

WOUND CLASS	Was antibiotics given?		
	Yes	No	Total
Clean	265(74.6%)	90(25.4%)	355(100%)
Clean contaminated	324(87.4%)	47(12.6%)	371(100%)
Contaminated			
Dirty			
Total	589(81.1%)	137(18.9%)	726(100%)

**Table 3:** Urological procedure and choice of antibiotics

Procedure	Choice of antibiotic given				
	Ceftriaxone alone	Metronidazole alone	Gentamycin alone	Ceftriaxone+Metronidazole	Others
DVU+CYSTOSCOPY	56.3%	6.4%	3.3%	32.8%	1.2%
URETHROPLASTY	51%	8.6%	7.4%	28.4%	4.6%
PROSTATECTOMY	56.5%	6.3%	14.2%	21.7%	1.3%
NEPHRECTOMY	10%	8%	3.6%	75.6%	2.8%
HYPOSPADIAS REPAIR	54.3%	12.6%	2.6%	28.7%	1.9%
OTHER(TCB,BSO,SPC,CYSTECTOMY)	48.4%	7.2%	4.8%	38%	1.8%
**Average percentage**	**46.1%**	**8.2%**	**6%**	**37.5%**	**2.3%**

## Discussion

In this study we found that urological surgeries are predominantly done in male with a wide range of ages. Most of our patients 589 (81.1%) were given antibiotics regardless of the type urological surgeries this is similar to the findings of others studies in Europe, United states and local studies that addresses the use of antibiotic prophylaxis in urologic interventions. Contrary to the recommendation from others studies that Antibiotics for most urologic interventions having only moderate to low evidence for the use of antibiotic prophylaxis, with the exception of TURP and prostate biopsy. Strong evidence supports the use of short-term prophylaxis for TURP, and this evidence is moderate to high for prostate biopsy. The main point of consideration when assessing the benefit of antibiotic prophylaxis is what to consider as a favourable outcome. In our study Eighty seven point four percent of clean contaminated urological surgeries were given prophylactic antibiotics also those who had clean urological surgeries 265 (74.6%) were given antibiotics [1, 3–5, 12, 17]. Our study did not explore on why patients were given antibiotics uphazardly but probably clinicians’ thoughts are to decrease of post intervention bacteriuria, or decrease of symptomatic UTIs or other infectious complications’ While the aim of preventing symptomatic UTIs and other serious infectious complications seems evident, the need to prevent asymptomatic bacteriuria remains questionable. Asymptomatic bacteriuria is often of no clinical importance and resolves spontaneously in many cases as the findings of other studies elsewhere [1, 3–11, 13, 18]. This being a retrospective study, we could not asses the outcomes of those patients post urological intervention therefore we could not find high evidence supporting the use of antibiotic prophylaxis in urologic interventions to prevent complications such as UTI including in those who had TURP done as it was found out in other studies that no enough evidence supports the systematic use of antibiotic prophylaxis to prevent UTIs in the rest of the procedures. However, when performing case notes review, we realized not only that variations in duration, antibiotic agent, or dose of what was considered “antibiotic prophylaxis” existed, but also that variables of importance in some patients who were discharged with urethral catheters or stents were insufficiently explored as those increases the risk of a post-operative infectious complication and, therefore, the need for an adequate antibiotic prophylaxis even in cases of low evidence for benefit. Still, good clinical practice should drive the decision in that circumstance [5, 10, 19, 20]. We found that Ceftriaxone was the commonly given antibiotic regardless of the type of urological surgery done similarly to the findings of other studies whereby overuse of third generation cephalosporin was noted, from this clinicians should keep in mind that antibiotic prophylaxis is only one of the various measures to prevent post-intervention infectious complications. Antibiotic prophylaxis cannot compensate for inadequate operative care, and, therefore, general recommendations for prevention of surgical site infections should be followed [1, 3–7, 21, 22].

## Conclusion

Antibiotic use is still a challenge at our hospital. Prolonged use of prophylactic antibiotics beyond five days was the main finding. Ceftriaxone was the most given antibiotic regardless of the urological surgery done and its level of contamination. Antibiotic stewardship needs to be addressed by adhering to antibiotics use guidelines and this will increase the quality of care and at the same time reduce both costs and the development of microbial resistance. Further research is needed because of lack of evidence that those patients with increased risk for infectious complications should receive antibiotic prophylaxis and why do clinicians give antibiotics empirically.
